# Editorial: Interaction of Biomolecules and Bioactive Compounds With the SARS-CoV-2 Proteins: Molecular Simulations for the Fight Against Covid-19

**DOI:** 10.3389/fmolb.2022.950891

**Published:** 2022-06-22

**Authors:** Mattia Falconi, James Leland Olds, Arvind Ramanathan

**Affiliations:** ^1^ Department of Biology, University of Rome “Tor Vergata”, Rome, Italy; ^2^ Schar School of Policy and Government, George Mason University, Fairfax, VA, United States; ^3^ Argonne National Laboratory (DOE), Lemont, IL, United States

**Keywords:** SARS-CoV-2, mpro protease, spike protein, computational methods, molecular simulation and docking

The Covid-19 pandemic, which we are still experiencing, has fostered the interest and study of all researchers worldwide who have made their expertise available to contribute to solving this global problem (Sharma et al., 2021). Computational scientists were able to continue their research when the experimentalists often had to leave their laboratories to prevent the spread of this dangerous virus. This pandemic has shown how computational approaches (Śledź and Caflisch, 2018; Patel et al., 2020; Romeo et al., 2020) can help the understanding of the structural basis underlying possible coronavirus inhibition mechanisms and how they may contribute to accelerating the discovery of novel treatment methods (Gurung et al., 2021). Although the release of very effective vaccines has largely helped people control this disease and reduce its burden on the worldwide population, there is still a lack of effective, safe, and broad-spectrum antiviral drugs to treat infected patients and stem future generations epidemics.

Thanks to the solution of the molecular structures composing the SARS-CoV-2 virion (Arya et al., 2021), many are the targets offered to molecular simulators who have designed various classes of molecules, peptides, or have selected proteins and antibodies to stem the spread of this threatening coronavirus.

This Special Issue collects computational research having two main coronavirus proteins as a target that allow and promote the SARS-CoV-2 infection, i.e., the main protease of the virus (Mpro) and the Spike glycoprotein.

Some of the collected papers deal with virtual screening applications associated with *in silico* or/and experimental validation of natural compounds or peptides that target the Mpro protease.

This target has been chosen to block the viral proteins processing. During host cell infection, the viral genome acts as messenger RNA. It directs the synthesis of two large polyproteins (pp1a and pp1ab), containing small proteins necessary to produce new viral particles inside infected cells. This set of proteins includes a replication/transcription complex, several structural proteins needed to build virions and two proteases (Wu et al., 2020; Zhou et al., 2020). The proteases play an essential role in cutting the two large polyproteins into smaller functional proteins. The SARS-CoV-2 main protease Mpro weighs 33.8 kDa and makes the most cuts. Mpro, essential for viral replication and absent in human cells, represents an optimal target for developing new antiviral drugs: blocking its functions would be lethal for the virus but safe for humans.

In this regard, to disable the SARS-CoV-2 Mpro activity, Piplani et al. carried out a computational repurposing of a series of drugs and natural products to be used as potential novel COVID-19 therapies; Cayona and Creencia tested the inhibitory potential of phytochemicals from the plant *Euphorbia hirta L.*; Kumar et al. evaluate the potential as inhibitors of natural alkaloids from Jadwar (*Delphinium denudatum*), and finally, Manivannan et al. evaluate the clove phytochemicals, a traditional natural therapeutic that comprises important bioactive compounds, as possible antiviral drug candidate targeting Mpro. The study has been carried out using molecular docking, molecular dynamics simulation and pharmacokinetic profiling. Interesting results have been obtained by Hernández González et al. designing and suggesting D-peptides as Mpro inhibitors. These authors use MM-GBSA free energy calculations, molecular dynamics (MD) simulations, and *in vitro* enzymatic assays of the four top-scoring D-tetrapeptides, all of which caused 55–85% inhibition of Mpro activity, thus highlighting the suitability of the devised approach.

The other section of this Special Issue turns to the other main research target i.e., the Spike glycoprotein (S) of the virus.

According to a key-lock model, viruses continuously evolve the proteins on their surface to enhance the interaction with the receptors on the cells and enter them more efficiently. This is also the case of SARS-CoV-2 Spike glycoprotein (the key) and the human Angiotensin Converting Enzyme 2 receptor (hACE2, the lock).

Spike protein is one of the most interesting and studied proteins that contribute to the binding with the host receptor and viral pathogenesis. Spike decorates the virus surface and is responsible for the viral surface corona appearance (Zhou et al., 2020). The virus uses this protein as a key to enter host cells (Tortorici et al., 2019). It acts by binding the receptor on target cells, inducing endocytosis of virions, catalyzing the fusion between cell and viral membranes and ensuring the entry of viral genomic RNA into the cytoplasm of cells. Spike protein is also the main target of the immune system, activating it and inducing the production of antibodies. For this reason, it is considered the primary target of antiviral drugs and vaccines, constituting a rich source of helpful information for the design of molecules able to inhibit its function and, therefore, potentially usable as therapeutic treatments.

In this regard, to explore effective inhibitory peptides against the Spike RBD of SARS-CoV-2, Biswas et al. have applied molecular docking and MD techniques on 23 antimicrobial peptides selected from literature. The obtained computational insights helped to understand the decrease in binding affinity of biliverdin with Spike caused by the R190K and N121Q mutations. Fung et al. have systematically investigated the RBD variants that markedly destabilize the binding to six neutralizing antibodies through in-depth mutational scanning; and finally Othman et al., using structural analysis and microscale accelerated MD, have explored the possibility of Spike Protein to bind integrins, proposed as host receptors for SARS-CoV-2, through the Arg-Gly-Asp (RGD) motif of the RBD.

In conclusion, deciphering the structure of critical virus constituents (Arya et al., 2021) has permitted computational researchers to apply their techniques and work to hypothesize a molecular solution to the pandemic problem. The experimental world has also greatly benefited from computational suggestions, discovering the usefulness of the simulation techniques.

We expect that this Special Issue will be helpful to experimentalists and clinicians and that it will further stimulate the use of novel and low-cost molecules to counteract the SARS-CoV-2 virus.
